# An early feasibility study for neurological devices: The ARCTRAN study

**DOI:** 10.1007/s10072-025-08367-5

**Published:** 2025-07-18

**Authors:** Francesco Bove, Giulia Di Lazzaro, Martina Petracca, Maria Rita Lo Monaco, Diego Ricciardi, Francesca Di Caro, Serena Fragapane, Silvia Giovannini, Chiara Iacovelli, Letizia Castelli, Assunta Bianco, Irene Scala, Aldobrando Broccolini, Pietro Caliandro, Domenica Le Pera, Carmen Furno, Giovanni Arcuri, Guerino Massimo Oscar Fares, Federico Spandonaro, Barbara Polistena, Paolo Calabresi, Anna Rita Bentivoglio

**Affiliations:** 1 Neurology Unit, Fondazione Policlinico Universitario A. Gemelli IRCCS, Rome, Italy; 2 Department of Neuroscience, Università Cattolica del Sacro Cuore, Largo Francesco Vito 1, Roma, 00168 Italia; 3 Geriatrics Unit, Fondazione Policlinico Universitario A. Gemelli IRCCS, Rome, Italy; 4 Department of Geriatrics and Orthopaedics, Università Cattolica del Sacro Cuore, Rome, Italy; 5 UOS Riabilitazione Post-Acuzie, Fondazione Policlinico Universitario A. Gemelli IRCCS, Rome, Italy; 6 Department of Emergency, Anaesthesiology and Intensive Care Medicine, Fondazione Policlinico Universitario A. Gemelli IRCCS, Rome, Italy; 7 IRCCS San Raffaele Roma-Pisana, Rome, Italy; 8 Health Technologies Unit, Fondazione Policlinico Universitario A. Gemelli IRCCS, Roma, Italy; 9 Università Roma Tre - Dipartimento di Scienze della formazione - Centro di ricerca Innovazione & Salute, Rome, Italy; 10 Università degli Studi di Roma Tor Vergata, Rome, Italy; 11 Centro per la Ricerca Economica Applicata in Sanità (C.R.E.A. Sanità), Rome, Italy

**Keywords:** Telerehabilitation, Early feasibility study, Parkinson’s disease, Multiple sclerosis, Stroke

## Abstract

**Introduction:**

An Early Feasibility Study (EFS) is an exploratory clinical investigation of a device to optimize its design through iterative feedback loops during early clinical experience. The ARCTRAN study is an EFS aimed at analyzing efficacy, safety and adherence to the home-rehabilitation device-based (ARC Intellicare) program compared to a paper-based exercise protocol in patients with Parkinson’s disease (PD), Multiple Sclerosis (MS) and stroke.

**Materials and Methods:**

At baseline (T0), patients of each group were randomly divided into two arms, with a 1:1 ratio: an ‘interventional’ group received ARC Intellicare and a ‘control’ group received a paper-based rehabilitation protocol, both consisting of three 60-minute sessions/week for 8 weeks. Each patient was evaluated by means of clinical scales, gait analysis and stabilometry at baseline (T0) and after the rehabilitation (T2).

**Results:**

All patients (*n* = 90, 30 per group) showed a significant improvement in clinical scales between T0 and T2. Stroke patients showed a bigger gait improvement in the ARC Intellicare arm, measured both by clinical scales (Tinetti gait *p* = 0.01) and gait analysis parameters. In PD group, only gait analysis parameters recorded a significant improvement in the active arm.

**Discussion:**

We demonstrate the safety of ARC Intellicare for home-based rehabilitation in patients with chronic neurological conditions, with high adherence levels. Moreover, in stroke group, it produced more significant improvements of gait compared to paper-based protocol. In the global scenario of the EFS projects promoted by the Italian Ministry of Health, this study is a starting point to conduct this type of studies in Italy.

## Introduction

An Early Feasibility Study (EFS) is an exploratory clinical investigation of a device early in the development phase, usually in a small number of patients. It mainly aims at evaluating the device design concept concerning clinical safety and device functionality, thus optimizing the device design through iterative feedback loops during early clinical experience. The American Food and Drug Administration (FDA) has developed an EFS program to increase access for patients to potentially beneficial technologies and to support device innovation, making the approval process timelier. In 2013, the guidance on “Investigational Device Exemptions (IDEs) for Early Feasibility Medical Device Clinical Studies” was published in US [1]. The 2017/745 Medical Device Regulation (MDR) of European Union (EU) [2], makes the regulatory framework for medical device in Europe; this Regulation sets high standards of quality and safety for medical devices by ensuring, among other things, that data generated in clinical investigations are reliable and robust and that the safety of the subjects participating in a clinical investigation is protected.

The rules on clinical investigations should be in line with well-established international guidance in this field, such as the international standard ISO 14155:2011 on good clinical practice for clinical investigations of medical devices for human subjects. In addition, the rules should be in line with the most recent version of the World Medical Association Declaration of Helsinki on Ethical Principles for Medical Research Involving Human Subjects.

As the manufacturers shall establish and update a clinical evaluation plan, which shall include also clinical development plan indicating progression from exploratory investigations, such as first-in-man studies, feasibility and pilot studies (ANNEX XIV MDR), the Ministry of Health declared in 2019 a public call, inspired to US EFS program, to test such model in Italian Context. The public competition was awarded by a temporary grouping of three Italian Units leading by Fondazione Policlinico Universitario Agostino Gemelli IRCCS (FPG, Rome, Italy). The grouping consists of FPG itself, which was responsible for the wall project and oversaw the clinical aspects of the project, Inter-departmental Research Centre for Political-Constitutional Studies and Comparative Legislation (CRISPEL, Rome, Italy), which dealt with the legislative aspects, Centre for Applied Health Economics (C.R.E.A. Sanità, Rome, Italy), which developed the economic aspects. This project founded by Italian Ministry of Health led to design and perform an EFS regarding a device of telerehabilitation (ARC Intellicare) in neurological disorders with motor disability. The clinical Investigation was submitted to Competent Authority according MDR rules [3].

For chronic neurological conditions, the access to in-person rehabilitation is often limited by several factors, such as reduced available economical resources by the Health Care System and difficulties of patients reaching the Center due to motor disability. It has been demonstrated that the interruption of rehabilitation programs increases patients’ need for assistance and the risk of falling, with heavy economic consequences for the Health Care System and a burden on socio-family management [4]. From a public health perspective, there is therefore an unmet need for continuous rehabilitation, given that chronic neurological pathologies significantly worsen during periods of inactivity following suspension of the rehabilitation intervention [5]. In the last 5 years, the available literature has demonstrated that, in terms of clinical effectiveness, telerehabilitation is comparable to in-person rehabilitation of patients with a previous stroke, in particular as concerns the recovery of global motor function, Activities of Daily Living (ADL) and independence [6]. Recent reviews on patients suffering from neurodegenerative disorders suggested that telerehabilitation may be effective in maintaining and/or improving some motor symptoms, including balance and walking, non-motor symptoms and quality of life [7]. Moreover, telehealth interventions appear to be beneficial in the long term and satisfying for patients and healthcare professionals [8].

ARC Intellicare is a new class I medical device, undergoing pre-market clinical investigation, which allows patients to follow a home rehabilitation program, with the possibility of remotely monitoring them. The device consists of a set of 5 inertial sensors inserted in special supports, a tablet with dedicated application, and a charging station. It allows clinicians to customize the rehabilitation program from a wide library of physical exercises and provides real-time feedback during the execution of motor and cardio-respiratory tasks, by means of wearable sensors and advanced intelligence algorithms artificial, so ensuring a higher compliance to home rehabilitation. Two previous uncontrolled small clinical studies tested the use of ARC Intellicare in patients affected by neurological disorders. In a multicenter study on post-stroke patients (ARCANGEL, ClinicalTrials.gov Identifier: NCT03787433), the use of ARC Intellicare was safe, without adverse events related to the use of the device, and high adherence to rehabilitation was shown, above all in the subgroup of patients with greater disability and greater competence in the use of technology. In another single-centre study on patients suffering from Parkinson’s disease and post-COVID-19 condition (RICOMINCIARE, ClinicalTrials.gov Identifier: NCT05074771), the adherence to rehabilitation program was above 75% for both motor and breathing exercises, without adverse events [9]. To date, further data are needed to support the use of ARC Intellicare in clinical practice in neurological disorders.

The aim of the present study is to evaluate efficacy and safety of ARC Intellicare in different neurological disorders with motor disability, and to assess the adherence of patients to the telerehabilitation protocol proposed.

## Materials and methods

### Study design and population

The ARCTRAN (ARC Intellicare TeleRehabilitation for Neurological Disorders) is a single-centre prospective randomized controlled early feasibility study, designed to test the feasibility of home rehabilitation with ARC Intellicare in patients with chronic neurological conditions associated to mild to moderate disability and thus requiring motor rehabilitation.

The study was conducted at Fondazione Policlinico Universitario Agostino Gemelli IRCCS in Rome under the coordination of Neurology Unit. The protocol was approved by the local Ethics Committee (ID5135) and complied with the Declaration of Helsinki and Good Clinical Practice (GCP) guidelines.

The study population enrolled in ARCTRAN comprised patients with 3 different neurological disease:

- Parkinson’s disease (PD);
- Relapsing-Remitting or Progressive Multiple Sclerosis (MS);
- Stroke with stabilized motor outcomes (stroke).

Each participant was randomly assigned to two arms, with a 1:1 ratio: an ‘interventional’ group received the ARC Intellicare device and a ‘control’ group received a paper-based rehabilitation protocol with the same training program.

### Inclusion and exclusion criteria

Inclusion criteria were: a diagnosis of Parkinson’s Disease according to MDS criteria [10] with at least 3 years disease duration and a Hoehn and Yahr (H&Y) staging from 1 to 3 [11], stable therapy in the last month, aged ≥ 50 and ≤ 75 years (group A); a diagnosis of relapsing-remitting or progressive Multiple Sclerosis, according to McDonald 2017 criteria [12] with an Expanded Disability Status Scale (EDSS) ≥ 3,5 and ≤ 6,5 [13], aged ≥ 18 and ≤ 60 years (group B); stroke occurred in the previous 12 months, with stabilized outcomes, a modified Rankin Scale (mRS) from 2 to 3 [14], aged ≥ 50 and ≤ 75 years (group C);

Exclusion criteria were: Mini Mental State Examination (MMSE) < 24 [15]; cortico-steroid therapy or initiation of a new disease-modifying drug (DMD) for MS in the three months prior to the enrolment; seizures or recent history of vertigo and/or falls; Tinetti Balance Scale < 19 [16]; active and/or unstable comorbidities; symptomatic orthostatic hypotension; severe orthopedic comorbidity.

### Visits timeline and study intervention

Each visit was performed by a multidisciplinary team; for PD group, the assessments were carried out in ON pharmacological conditions (at the best motor condition).

**Fig. 1:**
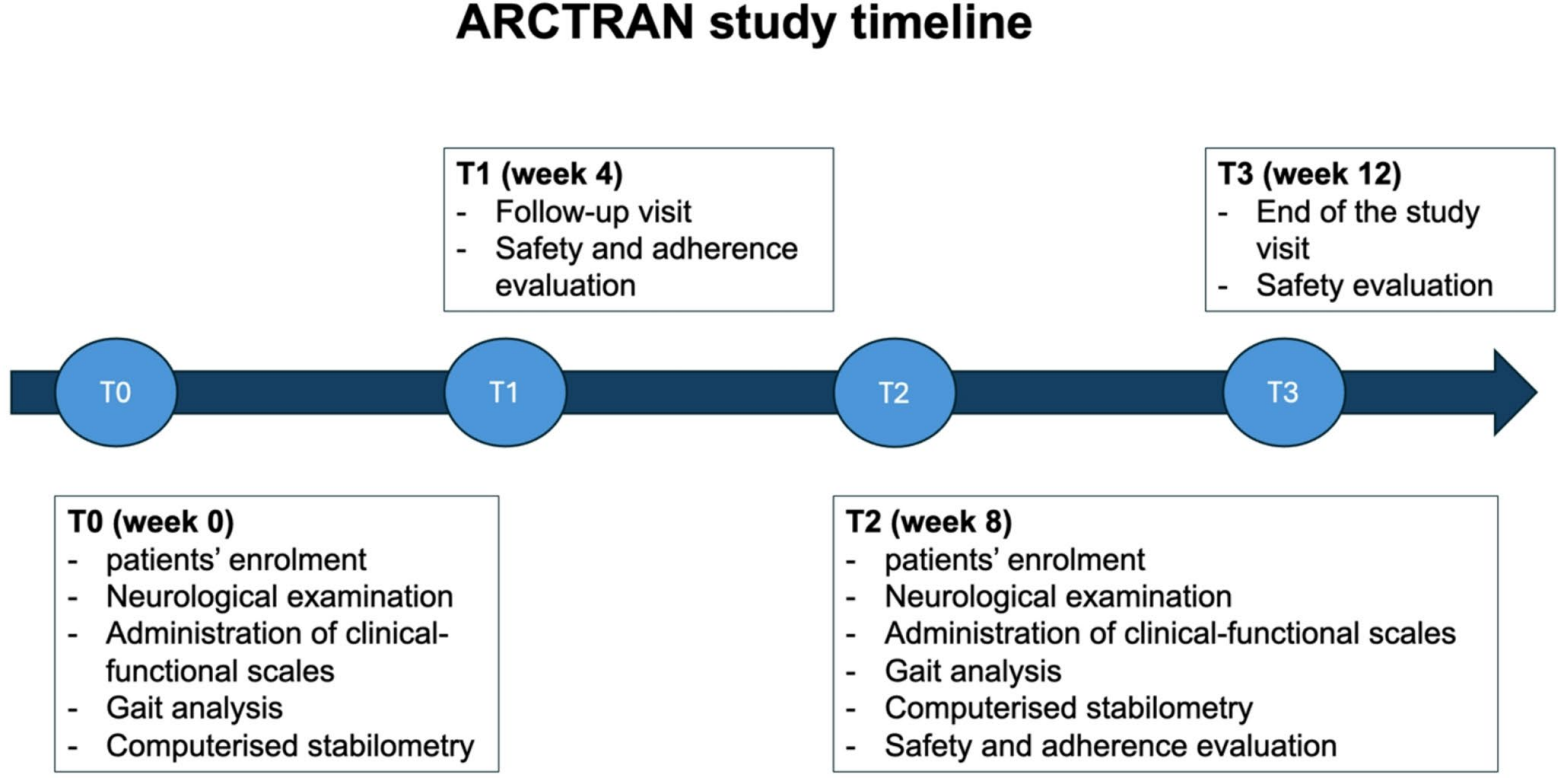
ARCTRAN study timeline

Each patient received 4 visits: baseline (T0), after 4 weeks (T1), to check for adherence and adverse events, after 8 weeks, at the end of the rehabilitation protocol (T2), and after 12 weeks (T3), for the end-of-study visit (Fig. 1).

At T0, patients were specifically instructed on the exercises to perform at home. After a joint example session, the subjects in the ARC Intellicare group were called in for testing the use of the device and for its delivery; the subjects in the control group were called in for evaluation and sharing of the rehabilitation plan proposed by the therapist, with the delivery of the paper-based rehabilitation program. The rehabilitation therapists drew up a uniform treatment plan for each pathology, based on the exercises available in the ARC Intellicare platform. For the control group, this plan was translated into a paper-based rehabilitation protocol.

For each patient, the therapists prescribed a home exercise of 60-minute sessions for 3 days a week, with a total duration of 8 weeks. For each group, patients were randomly assigned to ARC-assisted home rehabilitation, or to paper-based rehabilitation program, with sheets containing exercise descriptions. Adherence to treatment program was self-assessed by weekly questionnaires (Patient Reported Outcomes) in both groups and, in the active arm, was remotely monitored through ARC Intellicare.

### Clinical assessment

The patients underwent a neurological examination at each visit, while at T0 and T2 patients were evaluated with the following scales, considered as the main outcome measures for the efficacy of rehabilitation programs.

For the fall risk analysis, patients underwent the Tinetti scale [16]. The Montreal Cognitive Assessment (MoCA) was used as an additional screening assessment for detecting cognitive impairment [17]. Psychiatric symptoms in particular mood disturbances were assessed by means of Beck Depression Inventory (BDI) and Beck Anxiety Inventory (BAI) [18, 19]. Fatigue was assessed by Fatigue Severity Scale (FSS) [20]. For quality of life evaluation, we performed the European Quality of Life Scale (EQ-5D-5 L) [21].

For each group were also utilized specific clinical scales.

For PD group, the following clinical scales were performed: 1) the MDS-Unified Parkinson’s disease rating scale (MDS-UPDRS) [22]; the Parkinson’s Disease Questionnaire (PDQ-39), a QoL questionnaire for Parkinson’s disease patients, consisting of 39 questions [23]. For MS group, the MS impact scale 29 (MSIS-29) [24] and the MS Quality of life scale (MSQol-54) [25] were carried out. For the stroke group, we assessed the degree of disability or functional independence by means of the modified Rankin scale (mRS) [14]; the Fugl-Meyer Assessment (FMA) [26].

For the assessment of muscle strength and physical function, patients performed the six-minutes walking test (6MWT) [27], the timed-up-and-go (TUG) [28] and the short physical performance battery (SPPB) under the therapist supervision [29]. To assess the disability status, we performed the Barthel Index (BI) [30].

### Gait analysis

Instrumental gait analysis was performed at T0 and T2 visits in the motion analysis laboratory, using a Smart D500 stereo-photogrammetric optoelectronic system (BTS S.p.A., Garbagnate Milanese, MI, Italy) consisting of 8-camera, two force platforms, a traditional 2-camera video recording system, a 16-channel freemg unit, a synchronization and processing system for the various signals. The execution of the evaluation consisted of system calibration, detection of anthropometric parameters, application of acquisition markers, tracking, and data processing. Signal recording was made possible by placing 22 markers on specific anatomical repere points, according to the Davis protocol [31]. The assessment involved each patient, supervised by a dedicated operator, being asked to walk barefoot along a 6-meter path at his or her natural speed. This instrument allowed the ‘quantitative’ assessment of the characteristics of posture and normal or pathological walking through the extraction of the various parameters obtained from the movement of the body segments and joint excursions of interest in the three planes of space (x, y,z). The movement analysis was conducted by evaluating the spatio-temporal parameters, in particular stance phase, swing phase, cycle time, step length, cycle length and mean speed. These secondary outcome measures were computed at baseline (T0) and compared with the values determined at the end of treatment (T2). In order to analyze the dynamics during gait, platforms that returned the reaction force vector information (intensity, amplitude, direction and direction) during the stance phase within the gait cycle were used (see Guidelines of the Italian Society of Movement Analysis in the Clinic, SIAMOC).

### Stabilometry

Objective assessment of balance was performed at T0 and T2 with Hunova^®^ (Movendo Technology srl, Genoa, Italy), a robotic platform consisting of two electromechanical sensor platforms, one placed under the feet and the other under the seat, which allows the patient to be assessed while standing or sitting [32]. To detect trunk movements, the device works in tandem with a wireless 9-axis sensor (Inertial Movement Unit, or IMU) placed on the subject’s trunk. The IMU includes an accelerometer, gyroscope, and magnetometer [33]. Balance assessment was performed under static conditions, both with eyes open and closed, and stabilometric data were obtained from the analysis of Center of Pressure (COP) trajectories. The two variables COP eyes open and COP eyes closed were compared between T0 and T2 for all groups of patients and considered as secondary outcome measures.

### Adherence to the rehabilitative program and feasibility of the study

The adherence to the treatment proposed was measured by process indicators, such as attrition rate, and, for the interventional group, with other parameters obtainable through ARC Intellicare: number of sessions carried out/prescribed; time spent for execution; speed of execution of prescribed exercises; number of correct repetitions carried out/unit of time [9]. The home rehabilitation program adherence rate was a percentage of the number of sessions performed at home by the patient compared to the maximum number of prescribed rehabilitation sessions [34]. For both interventional and control groups, adherence has been obtained from questionnaires autonomously filled at home by patients with a weekly cadence. Comparison of adherence between the two groups has been based on such self-reported values. For the interventional arm, we also compared patient-reported and remote-controlled adherence in the three groups of patients (PD, SM, stroke). Feasibility of the device was measured by means of 2 indicators: System Usability Scale (SUS, score) [35] and patient satisfaction, assessed on a visual analogue scale (VAS).

### Safety

Safety was assessed in terms of the occurrence of adverse events (frequencies) by type (serious and non-serious, related/correlated and non-correlated to the use of ARC Intellicare). Fall diaries were given to participants for completion at each visit and collected at the next visit.

### Statistical analysis

Continuous variables were described as mean and standard deviation, whereas categorical variables were reported as count and percent. A descriptive analysis of the characteristics at baseline of the 3 patient groups (PD, MS, stroke) in the two treatment arms (ARC Intellicare versus control group) at baseline and after the treatment was performed. Owing to the limited sample size (15 subjects for each group), non-parametric tests were used to compare clinical-demographical characteristics among groups and within each group at baseline and after treatment. In particular, chi square was used to compare frequencies of categorical variables between groups, while a Mann-Whitney U test was used to compare continuous variables. A Wilcoxon signed-rank test was used to compare outcome measures at baseline and after treatment within each group. Statistical analyses were performed using SPSS software version 28. P-values smaller than 0.05 were considered significant. The p-values were adjusted for multiple comparisons using the Benjamini-Hochberg method for both primary and secondary outcomes of clinical efficacy. In particular, it was applied on the clinical scales assessing gait and balance (Tinetti Gait, Tinetti Balance, and Tinetti Total), gait analysis parameters (stance phase, swing phase, cycle time, step length, cycle length, and mean speed), and stabilometry measures (COP with eyes open and COP with eyes closed).

## Results

We enrolled 90 patients, 30 subjects with PD, 30 with MS and 30 with stroke. Clinical demographical data for each group are summarized in Table 1. To summarize, PD patients showed a mild parkinsonism (Hoehn & Yahr scale = 2 ± 0.5167), with a moderate impact of the disease on their quality of life (PDQ39 = 23.03 ± 17). With respect to the MS group, the study participants had an average disease duration of 9.27 ± 5.91 years with a mild disease severity (MSIS = 58.13 ± 16.73) and moderate impact on their quality of life (MSQOL-54 = 57.83 ± 29.129). Within the stroke group, the study participants had an average age of 64 years ± 7.5 and an average disease duration of 8 ± 3 months. They had a mild-moderate motor impairment (FMA = 177.59 ± 52.8; mRS = 2.23 ± 0.4).

**Table 1:** Demographic and clinical data of the patients at baseline (T0) and after two months of home rehabilitation (T2). B-H P value: Benjamini-Hochberg adjusted P value, (MoCA) Montreal cognitive Assessment, (SPPB) short physical performance battery, (BDI) Beck depression Inventory, (BAI) Beck anxiety Inventory, (EQ-5D-5 L) European quality of life scale, (SUS) system usability scal scale, (6MWT) Six-minutes walking test, (TUG) timed up and go test, (FSS) fatigue severity scale, (PDQ-39) parkinson’s disease Questionnaire, (H&Y) Hoehn & Yahr scale, (MDS-UPDRS) unified parkinson’s disease rating scale, (MSIS-29) multiple sclerosis impact scale 29, (MSQol-54) multiple sclerosis quality of life scale, (mRS) modified Rankin scale, (FMA) Fugl-Meyer assessment

	PD					MS					Stroke				
	**T0**		**T2**			**T0**		**T2**			**T0**		**T2**		
	**Mean**	**SD**	**Mean**	**SD**	**P value** **(B-H P value)**	**Mean**	**SD**	**Mean**	**SD**	**P value** **(B-H P value)**	**Mean**	**SD**	**Mean**	**SD**	**P value** **(B-H P value)**
Age	64	6				40.3	8.2				64	7.5			
Sex (M/F)	18/12					11/19					21/9				
Disease duration	5.33 y	3 y				9.27 y	5.91 y				8 m	3 m			
MoCA	25.8	4				25.33	3.49				23.27	3.4			
Tinetti balance	14.63	1	15.38	0.90	0.19 (0.28)	13.60	2.08	13.88	2.09	0.01 (**0.01**)	15.1	1.3	15.35	1.018	< 0.001 (**< 0.001**)
Tinetti gait	10.83	1	11.58	0.81	0.44 (0.44)	10.63	1.4	10.52	1.82	0.001 (**0.002**)	10.37	1.2	11.27	1.002	< 0.001 (**< 0.001**)
Tinetti total	25.47	2	26.96	1.22	0.024 (0.073)	24.23	3.05	24.40	3.73	< 0.001 **(< 0.001**)	25.47	2.0	26.62	1.699	< 0.001 (**< 0.001**)
SPPB	9.57	2	10.38	2	0.33	8.10	2.06	8.96	2.28	**< 0.001**	8.31	2.1	8.31	2.093	**0.001**
BDI	8.47	8	6.38	5.91	**< 0.001**	13.83	12.77	7.76	8.25	**0.008**	9.8	8.9	5.85	3.926	0.355
BAI	7	7	6.65	6.84	**< 0.001**	10.10	10.07	7.52	7.11	**< 0.001**	9.07	9.9	5.31	5.972	**< 0.001**
EQoL5	6.63	2	6.35	1.15	**< 0.001**	8.00	3.151	6.60	1.78	**0.002**	7.7	2.7	6.04	1.455	0.143
SUS	72.5	16	74.69	13.86	**< 0.001**	63.10	17.551	68.08	14.69	**< 0.001**	66.37	18.3	71.42	17.557	**0.017**
6MWT	512	806	560.85	136.15	**0.025**	434.93	102.69	480.88	118.20	**< 0.001**	434	105.3	494	123.2	**< 0.001**
TUG time	37	38	57.67	35.70	**0.021**	38.04	36.61	54.75	38.02	**0.001**	27.3	28.3	26.7	29.3	**0.016**
FSS	29.6	15	25.62	15.10	0.144	44.73	14.54	44.72	13.25	**< 0.001**	32.77	16.2	29.58	16.417	**< 0.001**
PDQ39	23.03	17	17	14.51	**0.036**	**---**	**---**	**---**	**---**		**---**	**---**	**---**	**---**	
Hoehn & Yahr	2	0.5167	2	0.54	**0.019**	**---**	**---**	**---**	**---**		**---**	**---**	**---**	**---**	
MDS-UPDRS I	11.47	5	8	4.06	**0.002**	**---**	**---**	**---**	**---**		**---**	**---**	**---**	**---**	
MDS-UPDRS II	8.37	4	7	4.21	**0.005**	**---**	**---**	**---**	**---**		**---**	**---**	**---**	**---**	
MDS-UPDRS III	21.87	8	18	7.22	**0.003**	**---**	**---**	**---**	**---**		**---**	**---**	**---**	**---**	
MDS-UPDRS IV	1.53	2	0.77	1.66	**< 0.001**	**---**	**---**	**---**	**---**		**---**	**---**	**---**	**---**	
MSIS	**---**	**---**	**---**	**---**		58.13	16.73	54.28	15.66	**< 0.001**	**---**	**---**	**---**	**---**	
MSQoL 54 tot	**---**	**---**	**---**	**---**		57.83	24.13	59.20	25.28	**< 0.001**	**---**	**---**	**---**	**---**	
mRS	**---**	**---**	**---**	**---**		**---**	**---**	**---**	**---**		2.23	0.4	1.85	0.675	**< 0.001**
FMA	**---**	**---**	**---**	**---**		**---**	**---**	**---**	**---**		177.59	16.1	195.69	14.38	**< 0.001**

Thirteen patients were lost at follow-up, 6 belonging to ARC Intellicare group (respectively 1 PD, 2 MS and 3 stroke) and 7 to paper-based exercise group (respectively 3 PD, 3 MS and 1 stroke). Eleven patients dropped out for unwillingness to continue the study, while two patients discontinued for medical reasons (atrial fibrillation with hospitalization and road accident with subarachnoid hemorrhage and clavicle fractures) unrelated to the medical device.

At T2, subjects from all groups, those who underwent rehabilitation with ARC Intellicare and those who followed the paper-based exercises, showed a significant improvement in several clinical scales assessing motor functions, mood disorders and quality of life (Table 1).

**Fig. 2:**
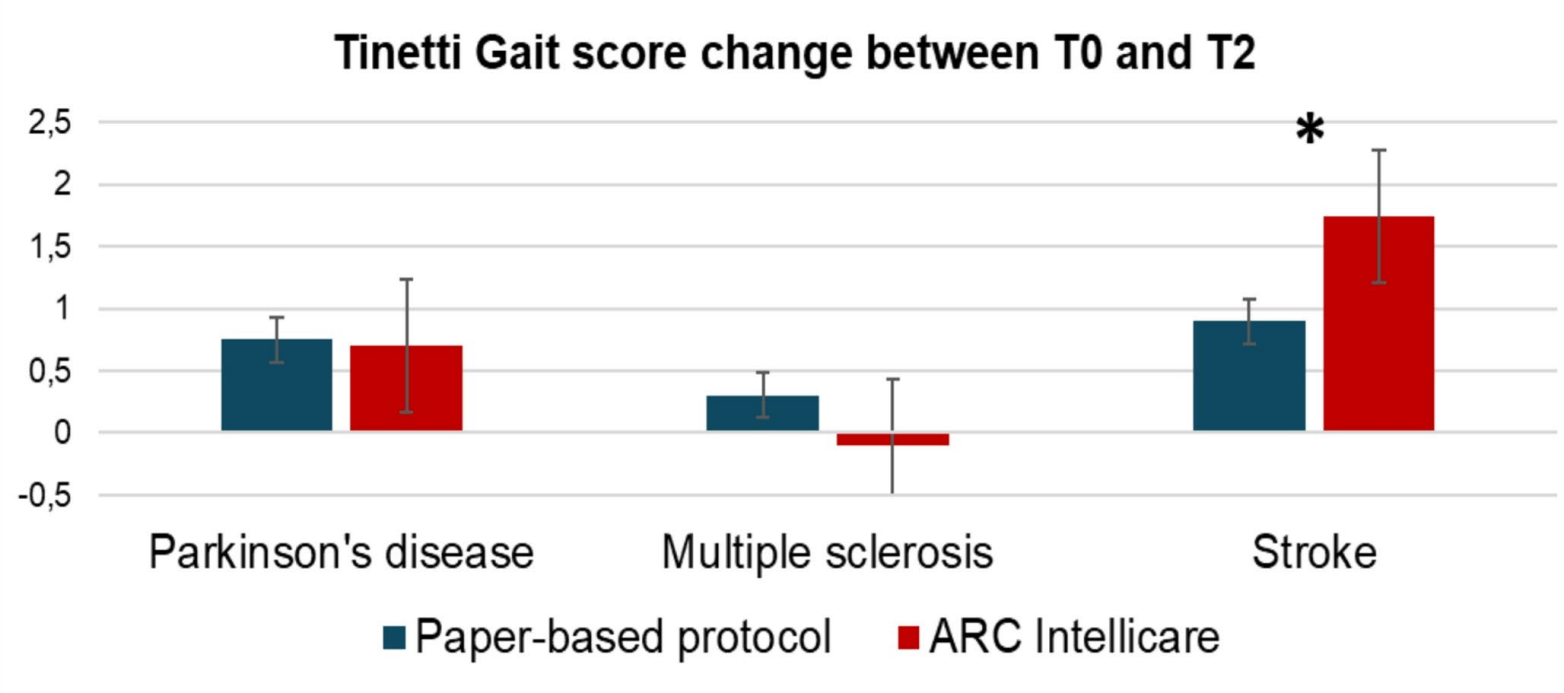
Tinetti gait score changes between T0 and T2 in the three groups

In the stroke population, gait improvement was significantly higher in the ARC Intellicare group when compared to the paper-based exercise arm (Tinetti gait score improvement ARC Intellicare versus paper-based home rehabilitation, + 1.74 versus + 0.9, *p* = 0.01, Fig. 2). Considering instrumental gait analysis (Table 2), intragroup analysis showed only in ARC Intellicare group a statistically significant improvement in cycle time of affected side (1.36 ± 0.15 s at T0 versus 1.19 ± 0.32 s at T2, *p* = 0.015) and unaffected side (1.37 ± 0.15 s at T0 versus 1.19 ± 0.32 s at T2, *p* = 0.009), and mean speed (0.74 ± 0.14 m/sec at T0 versus 0.84 ± 0.17 m/sec at T2, *p* = 0.039). Comparison between groups showed that cycle time of unaffected side more significantly improved in ARC Intellicare group (*p* = 0.047) (Table 2). In PD population, intragroup analysis showed that only in ARC Intellicare group some gait parameters showed statistically significant changes, such as cycle time of right side (1.16 ± 0.10 s at T0 versus 1.10 ± 0.09 s at T2, *p* = 0.032) and left side (1.16 ± 0.10 s at T0 versus 1.11 ± 0.09 s at T2, *p* = 0.041), and cycle length of right side (1.16 ± 0.10 m at T0 versus 1.21 ± 0.08 m atT2, *p* = 0.038) and left side (1.15 ± 0.10 m at T0 versus 1.20 ± 0.08 m at T2, *p* = 0.035). However, comparison between groups showed no statistically significant differences. In MS group emerged inconsistent results: step length of left side improved only in ARC Intellicare group (0.49 ± 0.12 m at T0 versus 0.53 ± 0.08 m at T2, *p* = 0.037), while cycle length improved only in the control group (on right side 1.09 ± 0.16 m at T0 versus 1.11 ± 0.18 m at T2, *p* = 0.025; on left side 1.08 ± 0.16 m at T0 versus 1.12 ± 0.17 m at T2, *p* = 0.021). Intergroup comparison showed no statistically significant differences between the ARC Intellicare group and the paper-based exercise group (Table 2).

**Table 2:** Gait analysis and stabilometry parameters in the three groups of patients, who underwent to home rehabilitation with ARC intellicare or with a sheet protocol, at baseline (T0) and after two months of home rehabilitation (T2). B-H P value: Benjamini-Hochberg adjusted P value. COP = Center of pressure; m = meters; s = seconds

	ARC Intellicare			Paper-based protocol			ARC Intellicare vs. Paper-based protocol *P* value (B-H *P* value)
	T0	T2	*P* value (B-H *P* value)	T0	T2	*P* value (B-H *P* value)	
PD							
Gait analysis							
Stance phase right (%)	60.55 ± 2.75	59.47 ± 2.77	0.055 (0.087)	60.74 ± 2.63	60.03 ± 1.84	0.835 (0.918)	0.045 (0.247)
Stance phase left (%)	60.79 ± 2.71	60.08 ± 2.78	0.387 (0.473)	60.61 ± 2.77	60.00 ± 1.84	0.025 (0.139)	1.0 (1.0)
Swing phase right (%)	39.45 ± 2.75	40.53 ± 2.77	0.066 (0.091)	39.26 ± 2.63	39.97 ± 1.84	0.921 (0.921)	0.045 (0.247)
Swing phase left (%)	39.21 ± 2.71	39.92 ± 2.78	0.803 (0.803)	39.39 ± 2.77	40.00 ± 2.31	0.04 (0.147)	1.0 (1.0)
Cycle time right (s)	1.16 ± 0.10	1.10 ± 0.09	0.003 (**0.032**)	1.13 ± 0.11	1.10 ± 0.11	0.012 (0.132)	0.416 (0.508)
Cycle time left (s)	1.16 ± 0.10	1.11 ± 0.09	0.015 (**0.041**)	1.13 ± 0.11	1.11 ± 0.11	0.169 (0.372)	0.311 (0.427)
Step length right (m)	0.53 ± 0.05	0.56 ± 0.04	0.032 (0.059)	0.52 ± 0.09	0.55 ± 0.07	0.102 (0.281)	0.101 (0.277)
Step length left (m)	0.54 ± 0.05	0.56 ± 0.04	0.026 (0.057)	0.52 ± 0.08	0.54 ± 0.07	0.307 (0.483)	0.018 (0.196)
Cycle length right (m)	1.16 ± 0.10	1.21 ± 0.08	0.01 (**0.038**)	1.14 ± 0.18	1.19 ± 0.17	0.398 (0.547)	0.22 (0.345)
Cycle length left (m)	1.15 ± 0.10	1.20 ± 0.08	0.006 (**0.035**)	1.13 ± 0.18	1.18 ± 0.16	0.654 (0.779)	0.132 (0.291)
Mean speed (m/s)	0.97 ± 0.12	1.06 ± 0.14	0.54 (0.594)	0.99 ± 0.23	1.05 ± 0.21	0.252 (0.463)	0.169 (0.31)
Stabilometry							
COP eyes open (cm)	36.88 ± 17.62	40.23 ± 77.13	0.48 (0.48)	49.40 ± 44.15	30.40 ± 11.81	0.839 (0.839)	1.0 (1.0)
COP eyes closed (cm)	68.59 ± 23.10	21.59 ± 22.49	0.291 (0.583)	73.13 ± 57.80	59.43 ± 23.03	0.323 (0.647)	0.42 (0.840)
MS							
Gait analysis							
Stance phase right (%)	63.11 ± 4.70	62.38 ± 4.62	0.339 (0.746)	64.43 ± 4.20	62.98 ± 4.30	0.014 (0.051)	0.219 (0.481)
Stance phase left (%)	63.95 ± 4.98	63.50 ± 4.21	1.0 (1.0)	62.53 ± 3.61	62.92 ± 4.50	0.703 (0.859)	0.092 (0.339)
Swing phase right (%)	36.89 ± 4.70	37.62 ± 4.62	0.563 (0.884)	35.57 ± 4.20	37.02 ± 4.30	0.014 (0.051)	0.128 (0.353)
Swing phase left (%)	36.05 ± 4.98	36.50 ± 4.21	0.906 (0.997)	37.47 ± 3.61	37.08 ± 4.50	1.0 (1.0)	0.781 (0.859)
Cycle time right (s)	1.31 ± 0.33	1.27 ± 0.23	0.069 (0.253)	1.29 ± 0.15	1.25 ± 0.11	0.292 (0.402)	0.912 (0.912)
Cycle time left (s)	1.33 ± 0.38	1.27 ± 0.24	0.012 (0.064)	1.28 ± 0.14	1.25 ± 0.11	0.136 (0.214)	0.435 (0.684)
Step length right (m)	0.50 ± 0.11	0.53 ± 0.08	0.214 (0.589)	0.50 ± 0.08	0.51 ± 0.08	0.858 (0.944)	0.01 (0.105)
Step length left (m)	0.49 ± 0.12	0.53 ± 0.08	0.003 (**0.037**)	0.50 ± 0.06	0.51 ± 0.09	0.114 (0.209)	0.236 (0.483)
Cycle length right (m)	1.11 ± 0.19	1.12 ± 0.17	0.768 (0.939)	1.09 ± 0.16	1.11 ± 0.18	0.005 (**0.025**)	0.435 (0.684)
Cycle length left (m)	1.10 ± 0.20	1.12 ± 0.17	0.47 (0.861)	1.08 ± 0.16	1.12 ± 0.17	0.002 (**0.021**)	0.019 (0.106)
Mean speed (m/s)	0.86 ± 0.24	0.90 ± 0.20	0.677 (0.931)	0.84 ± 0.17	0.87 ± 0.17	0.04 (0.089)	0.435 (0.684)
Stabilometry							
COP eyes open (cm)	40.12 ± 16.12	34.07 ± 10.45	0.65 (0.65)	50.14 ± 23.17	51.53 ± 21.86	0.468 (0.937)	0.251 (0.503)
COP eyes closed (cm)	89.15 ± 54.58	68.17 ± 34.53	0.155 (0.311)	103.67 ± 33.75	104.46 ± 45.84	0.988 (0.988)	1.0 (1.0)
Stroke							
Gait analysis							
Stance phase affected side (%)	62.11 ± 2.76	62.45 ± 2.13	0.583 (0.712)	62.02 ± 2.48	62.76 ± 4.08	0.609 (0.67)	0.396 (0.436)
Stance phase unaffected side (%)	64.34 ± 3.36	63.64 ± 2.61	0.058 (0.091)	63.85 ± 5.18	64.31 ± 7.16	0.044 (0.097)	0.022 (0.079)
Swing phase affected side (%)	37.89 ± 2.76	37.55 ± 2.13	0.655 (0.721)	37.98 ± 2.48	37.24 ± 4.08	0.96 (0.96)	0.229 (0.315)
Swing phase unaffected side (%)	35.66 ± 3.36	36.36 ± 2.61	0.027 (0.074)	36.15 ± 5.18	35.69 ± 7.16	0.291 (0.457)	0.01 (0.053)
Cycle time affected side (s)	1.36 ± 0.15	1.19 ± 0.32	0.003 (**0.015**)	1.33 ± 0.29	1.38 ± 0.48	0.019 (0.068)	0.065 (0.143)
Cycle time unaffected side (s)	1.37 ± 0.15	1.19 ± 0.32	< 0.001 (**0.009**)	1.33 ± 0.29	1.38 ± 0.50	0.012 (0.067)	0.004 (**0.047**)
Step lenght affected side (m)	0.46 ± 0.06	0.49 ± 0.05	0.035 (0.076)	0.44 ± 0.13	0.46 ± 0.13	0.027 (0.074)	0.793 (0.793)
Step lenght unaffected side (m)	0.47 ± 0.05	0.49 ± 0.06	0.045 (0.082)	0.45 ± 0.13	0.46 ± 0.14	0.094 (0.173)	0.048 (0.132)
Cycle length affected side (m)	1.00 ± 0.11	1.05 ± 0.13	0.843 (0.843)	0.97 ± 0.29	1.00 ± 0.29	0.389 (0.535)	0.157 (0.247)
Cycle lenght unaffected side (m)	1.00 ± 0.11	1.06 ± 0.13	0.481 (0.661)	0.97 ± 0.29	1.00 ± 0.30	0.468 (0.572)	0.092 (0.168)
Mean speed (m/s)	0.74 ± 0.14	0.84 ± 0.17	0.011 (**0.039**)	0.77 ± 0.30	0.81 ± 0.31	0.004 (**0.049**)	0.322 (0.393)
Stabilometry							
COP eyes open (cm)	38.37 ± 13.02	41.82 ± 12.97	0.286 (0.286)	36.88 ± 17.73	38.40 ± 11.06	0.297 (0.594)	0.809 (0.809)
COP eyes closed (cm)	66.47 ± 36.47	76.87 ± 33.44	0.03 (0.061)	67.90 ± 33.99	62.44 ± 25.04	0.778 (0.778)	0.048 (0.096)

Regarding balance assessment with the Hunova^®^ robotic platform, neither intragroup analysis nor between group comparison showed statistically significant results in each population analyzed (Table 2).

**Fig. 3:**
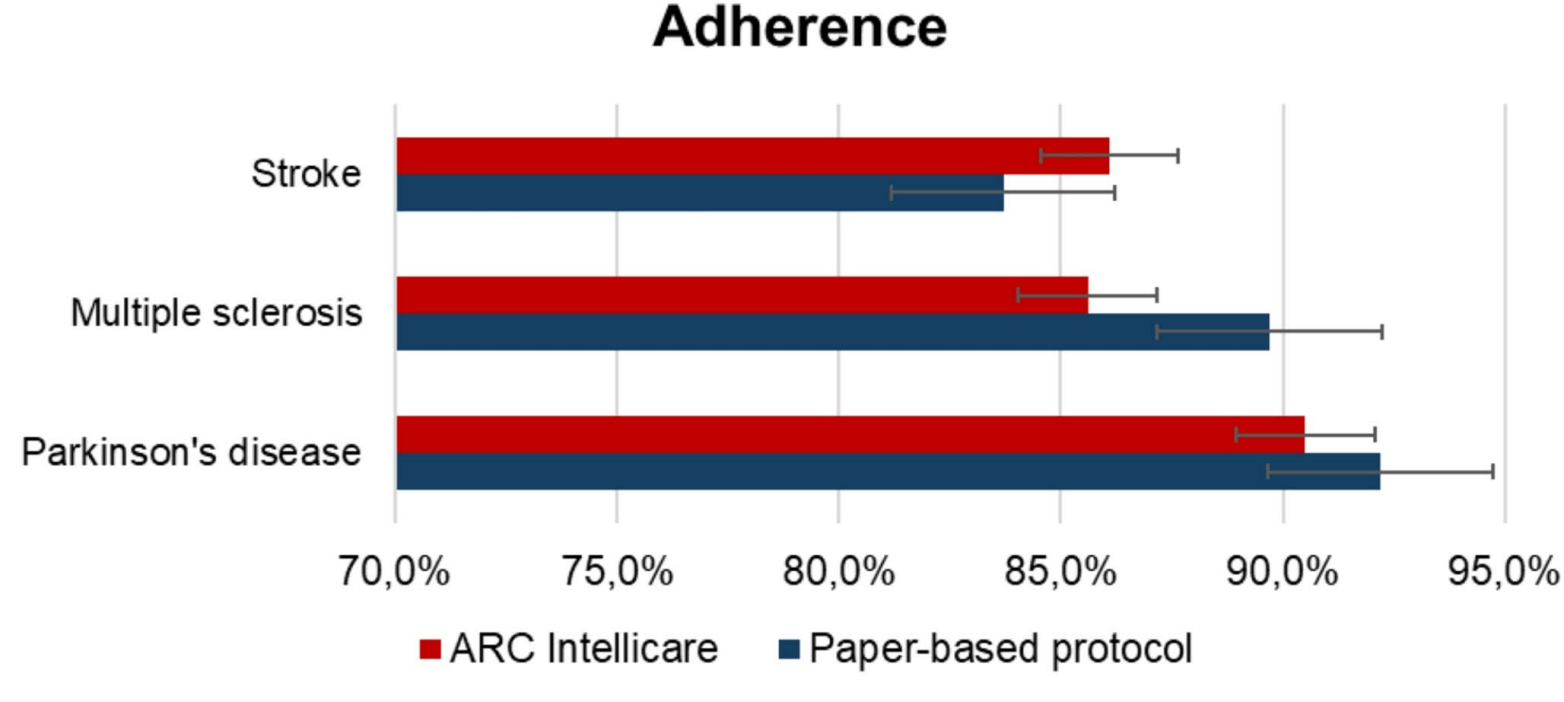
Patient-reported adherence to treatment in the three groups of patients

No significant adverse events were recorded, nor falls. Adherence was good for all groups and the device was found overall user-friendly. In particular, patient-reported adherence was higher than 80% in all groups with no significant differences (Fig. 3). When comparing patient-reported and remote-controlled adherence in the active arm, we found no significant differences in the three groups (in PD group 90.5% patient-reported adherence versus 91.1% remote-controlled adherence; in MS group 85.6% patient-reported adherence versus 84% remote-controlled adherence; in stroke group 86.1% patient-reported adherence versus 85.2% remote-controlled adherence). SUS was overall higher than 80%, with PD group scoring 89.29 at T2, MS group 91.35 and stroke group 81.82. VAS was 90.8% for the whole sample, scoring respectively 89.1%, 90.7% and 92.3% for the three groups (PD, MS and stroke respectively).

## Discussion

The main results of this EFS are the safety and the effectiveness of ARC Intellicare, compared to a paper-based protocol of home rehabilitation. Notably, with respect to the efficacy, only in the group of patients suffering from stroke the device-guided telerehabilitation was associated to better outcomes. In this population, after the period of physical therapy, ARC Intellicare provided higher improvement of gait than paper-based protocol. Gait improvement was detected by both clinical scales (Tinetti test) and objective assessment (gait analysis). These findings support the results of previous studies, which reported the effectiveness of device-assisted telerehabilitation in recovering gait after stroke [36, 37]. In our study, the better outcomes in the ARC Intellicare groups were appreciable on a complex motor function, as gait, more than segmental muscle strength or coordination, probably due to the greater need for feedback control in gait rehabilitation. As widely documented in literature, walking difficulties are strictly related to falls after stroke [38], and physical therapy reduce the risk of falling [39], impacting on the quality of life of patients. In this EFS, we cannot specifically investigate the effect of telerehabilitation on falls prevention, as we did not recruit faller patients. Considering the benefits provided on gait, future studies with a long-term follow-up should be specifically designed to assess the effect of ARC Intellicare on falls following stroke.

On the other hand, comparing home rehabilitation with ARC Intellicare versus paper-based protocol in patients affected by PD and MS, we did not find significant differences in clinical scales. In PD patients, only in the active arm, we found an improvement of objective gait measurements (assessed by gait analysis) that may suggest a subclinical but still detectable beneficial effect of ARC Intellicare on gait rehabilitation in this population, as reported in a previous pilot study [9]. However, the absence of improvement in clinical scales calls for precaution when interpreting these findings. In MS patients, inconsistent results were detected by gait analysis and stabilometry in the active arm.

Globally, these results suggest that home rehabilitation guided by ARC Intellicare is more effective than home rehabilitation guided by a paper-based protocol mainly after acute neurological conditions, as stroke. In our study, we enrolled patients with an acute event occurred within one year, following the stabilization of motor outcomes. It is likely that, in this post-acute phase, the qualitative feedback offered by ARC Intellicare may provide a greater effectiveness of the physical therapy. On the contrary, in patients suffering from chronic neurological diseases, who already underwent previous rehabilitation cycles supervised by therapists, the benefit due to the qualitative feedback offered by device has lesser impact, as they were already trained in physical therapy. However, in chronic pathologies such as PD and MS, in which the benefits of continuous rehabilitation therapy has been documented [5], studies with a longer duration of treatment are needed to establish the potential usefulness of treatment with the device. Moreover, device-guided telerehabilitation may be considered in patients affected by chronic neurological conditions who do not have access to in-person rehabilitation [7, 8].

After physical therapy, in both arms of treatment, all the three groups of patients included in the study showed improvement in motor functions, mood and quality of life. This may be due to home rehabilitation itself, which provides several motor and non-motor benefits in patients affected by acute and chronic neurological diseases [7].

Another important result of this EFS is the safety of ARC Intellicare, as no device-related adverse event was reported in the whole patient’s sample. Globally, the patients expressed a high level of appreciation towards ARC Intellicare, with an average score of VAS higher than 90%, and a good usability of the device, with an average score of SUS over 70/100, recognized as an appropriate cut-off in literature [35]. Device usability is strictly related to adherence to treatment, as poor usability is one of the main causes of technological system abandonment [40] and should be carefully investigated in an early phase of development of a telerehabilitation device. In all groups of the study, without significant differences between the two arms of intervention, the adherence to treatment was higher than 80%, the suggested cut-off for an effective adherence to the rehabilitation program [34]. Although the treatment with ARC Intellicare does not appear to increase the rate of adherence to rehabilitation compared to treatment with paper-based protocol, it should be considered that adherence was also objectively measured by device in the active arm, whereas it was only self-assessed by the patients in the control arm. Since the adherence to home rehabilitation in neurological disorders varies from 30 to 70%, according to literature’s data [9], there was a possible overestimation of adherence in the control arm, thus highlighting the importance of an objective device-based monitoring during home rehabilitation.

This study has inevitable limitations. As an EFS, the sample size of each arm of treatment is low and the results should be validated in larger populations. A second limitation, which may have led to an underestimation of the clinical efficacy of ARC Intellicare versus the paper-based protocol, is that the control group underwent active home rehabilitation with a regular supervision by follow-up visits with expert physiotherapists. This is not the real standard of care, as usually patients do not perform home rehabilitation between different cycles of in-person physical therapy at a Center. However, we decided to propose an alternative rehabilitation to the control group for ethical reasons, as well as physical inactivity has been documented to be detrimental in chronic neurological conditions [5, 7]. A third limitation is the absence of an objective measurements of adherence to treatment in the control arm, as above discussed.

In the global scenario of the EFS project promoted by the Italian Ministry of Health, this study is a starting point to conduct this type of studies in Italy, in compliance with current European regulatory law [2]. In this pilot experience we traced a standardizable framework, with all the steps implied in the selection of a device, a model for structuring the clinical protocol, and the professional figures involved at each phase of the study. Moreover, we demonstrated that cooperation among clinicians, biomedical engineers, device industry and regulatory authorities, is feasible the Italian context and ensures that every aspect of the device - from design to clinical use - is optimized to meet the needs of patients and the expectations of healthcare professionals. Indeed, the feedback provided by patients and clinicians during the study allowed to the manufacturer to optimize ARC Intellicare in both hardware and software components. Regarding the hardware: the recharging phase of the sensors was improved by optimizing the matching between the sensors and the connector components of the charging station; the Instructions For Users (IFU) were updated to clarify some steps related to the cleaning procedure of hardware components. Regarding the software, additional features have been developed and released, after the end of the study, based on the users’ feedback: in the updated version of the device, sensors pairing is required just at the beginning of the session, and the patient is supported thanks to the availability of visual and audio feedback. Finally, the inertial data collected during the ARCTRAN study allowed the manufacturer to improve its machine learning algorithm, retrain exercise models, increase their accuracy and release a new version of the machine learning module. This expanded collaboration is crucial to drive innovation in the field of medical devices, facilitating a faster development pipeline from concept to market. Although the project was conducted on a class I medical device, it provided valuable results, for the specific device evaluated and in a global view of public health. In the Italian context, future EFS are needed with more invasive devices (classes II and III), which are more challenging in terms of safety issues.

In conclusion, the results of this EFS indicate that ARC Intellicare is a safe and easily usable device by patients in home rehabilitation, with a good adherence rate. It provides improvement of motor function in patients affected by outcomes of acute neurological diseases, as stroke, while the clinical effects in chronic neurological disorders should be evaluated in studies with longer follow-up. The development of the EFS project in Italy will offer new possibilities for medical devices which potentially will improve diagnosis and treatment of neurological diseases.
